# HIV care cascade and sustainable wellbeing of people living with HIV in context

**DOI:** 10.1002/jia2.25259

**Published:** 2019-02-19

**Authors:** Hakan Seckinelgin

**Affiliations:** ^1^ Department of Social Policy London School of Economics and Politics (LSE) London United Kingdom

**Keywords:** Justice, ART, TasP, Policy, Public health, Human rights

Given the widely observed successes of treatment rollout, the central question for the future of international AIDS policies, is how to build and to support conditions that promote sustainable wellbeing for those with HIV, while also dealing with immediate HIV policy imperatives 1. Here I ask – does the HIV care cascade do this?

One of the central international AIDS policy frameworks currently is *Treatment as Prevention (TasP)* 2, 3. It aims to both provide antiretroviral treatment to those who need it and to tackle a fundamental challenge in the global fight against the disease: preventing further spread of HIV. While increased access to antiretroviral treatment globally has allowed more and more to live longer lives, a recent UNAIDS report shows it is hard to control the epidemic by controlling new infections. Notwithstanding increased access to treatment and despite the observed decline in new infections, 1.8 million people were “[still] infected in 2017”; these mostly included women in Africa and key populations and their partners globally 4.

*TasP* aims to tackle the problem through new policies and intervention strategies. Its goal is operationalized by the *global 90‐90‐90 targets* that are to be achieved by 2020 that aim to “end the AIDS epidemic by 2030” 5. The implementation mechanism of the policy is captured in the 90‐90‐90 label: “if 90% of all people living with HIV will know their HIV status and 90% of all people with diagnosed HIV infection will receive sustained antiretroviral therapy and 90% of all people receiving antiretroviral therapy will have viral suppression by 2020” this will mean that “at least 73% of all people living with HIV worldwide will be virally suppressed” and according to “modelling [this] suggests that achieving these targets by 2020 will enable the world to end the AIDS epidemic by 2030” (5, p. 2).

The implementation mechanism of the 90‐90‐90 targets is based on and uses the logic of the HIV care cascade. The logic is presented by Gardner at al. in their influential 2011 study reviewing HIV treatment and care in the US. They review the epidemiological data to “describe and quantify the spectrum of engagement in HIV care … and better understand how gaps in the continuum of HIV care affect virological outcomes in the United States” [6, p. 793]. This mapping exercise highlights gaps in the US system in terms of increasing HIV testing rates and keeping people in the care system once they are tested. This presents an analytical logic of the cascade by identifying suboptimal linkages within the “spectrum of engagement in HIV care” as “significant barriers” to achieving good treatment outcomes’ [6, p. 792]. They “posit that expanded testing and earlier treatment of HIV infection could markedly decrease ongoing HIV transmission” [6, p. 793]. The important basic insight in this analysis is that the potential of antiretroviral therapy to produce wellbeing and to achieve undetectable viral load is a function of how individuals living with HIV are located within the care system and provided support over time. The analytical lens of the care cascade provides a way to evaluate where the gaps are in the existing treatment, healthcare support for people with HIV 7, 8, 9, 10.

The emerging causal narrative presents a sequential *if then* logic that begins with a positive testing result and ends with viral suppression in individuals. It is this causal pathway, emerging from the data analysis that has become the policy model framing *TasP* as to how policy interventions should be implemented to achieve the overall policy outcome of stopping the epidemic through individuals with HIV achieving viral suppression. The 90‐90‐90 policy uses this logic as the mechanism for global policy implementation by linking each stage of the cascade with quantifiable policy targets to be achieved. This mechanism creates compartmentalized research and policy that aim at each stage to isolate and focus on those individuals who are identified as not engaging with the health system, so that at the end, once policies produce expected results at each stage, the policy is supposed to produce cumulatively the overall outcome of viral suppression at population level, and so ending the epidemic. UNAIDS states that “[T]hese new targets address progress along the HIV cascade engagement in care, measuring the degree to which programmes are meeting their ultimate goal of viral suppression” [5, p. 10].

Policy actors and researchers assume that in each policy context the gaps identified by the HIV care cascade will allow better policy targeting of different stages of the HIV experience. As a result, as observed in the 90‐90‐90 policy, the causal narrative of the cascade is used to frame the step‐by‐step policy directions to progressively achieve expected outcomes: viral suppression leading to decline in HIV spread. People living with HIV and their everyday lives are considered within the framing of this causal narrative. For policy success, those with HIV are expected to remodel their health behaviours both to become healthier and to be the agents of change within the stepped logic of the cascade. This approach compartmentalises peoples’ lives according to the needs of the policy, to achieve policy targets 11. The stability of the policy outcome, as a way of controlling the spread of the disease, thus relies on the ability of health system to turn interventions furthering viral suppression into normal ways of being and doing.

However, viral suppression is a process that needs to be reproduced by people in their everyday lives under dynamics that are typically different from the policy implementation contexts that are created and supported by international resources. These resources are also not available in perpetuity. The examination of conditions under which stable viral suppression can be sustained is not part of the care cascade model. The care cascade research and its dependent policies have little to say about how contexts of peoples’ lives inform their everyday health behaviour. In the end, both consider the causal factors operational in achieving the viral suppression to be similar to those causal factors that will make that outcome state stable and sustainable: in essence that individuals can maintain the state of viral suppression once achieved. But the sustainability of such stability in individuals’ lives relies on different dynamics and how these influence individual health practices 12, 13. These dynamics include personal choices, attitudes, interpersonal relations, infrastructure, access arrangements. By excluding these contextual social, economic and political factors, the HIV care cascade produces pragmatic policy orientations that fail to the capture conditions of sustainable wellbeing, without which viral suppression cannot be maintained in everyday lives of people with HIV.

HIV care cascade‐based policies are part of human rights commitments made by the global AIDS community to deliver resources. In this, independent of policy makers’ good intentions, peoples’ lives remain “precarious” 14. Beyond delivering human rights commitments, social justice considerations imply that these supply side policies ought to be evaluated in terms of the everyday experiences of those subject to the policies and in terms of the sustainability of their lives. Sen's consequentialist evaluations consider this to be central for judging the goodness and relevance of policies 15. Capability assessments are essential in relation to “the freedom to pursue well‐being.” Here freedom is about the choices people are able to make in order to live lives they want to live [16, p. 39]. By failing to engage in this, international policy actors could also be, from Pogge's 16 justice considerations, breaching their negative duties not to harm, because the sustainability of their policies is not guaranteed. Considering their resource base and their policy focus, HIV care cascade‐based policies arguably become part of the precariousness, rather than solving it and so, fall short in social justice considerations.

## Competing interests

None.

## Authors’ contributions

HS is responsible for all the entirety of the piece.
